# Supplementary material for: Machine learning models can identify individuals based on a resident oral bacteriophage family
Source: Front Microbiomes. 2024 Sep 3;3:1408203. doi: 10.3389/frmbi.2024.1408203 (PMC12993541; doi:10.3389/frmbi.2024.1408203)

#### **SI Figures**


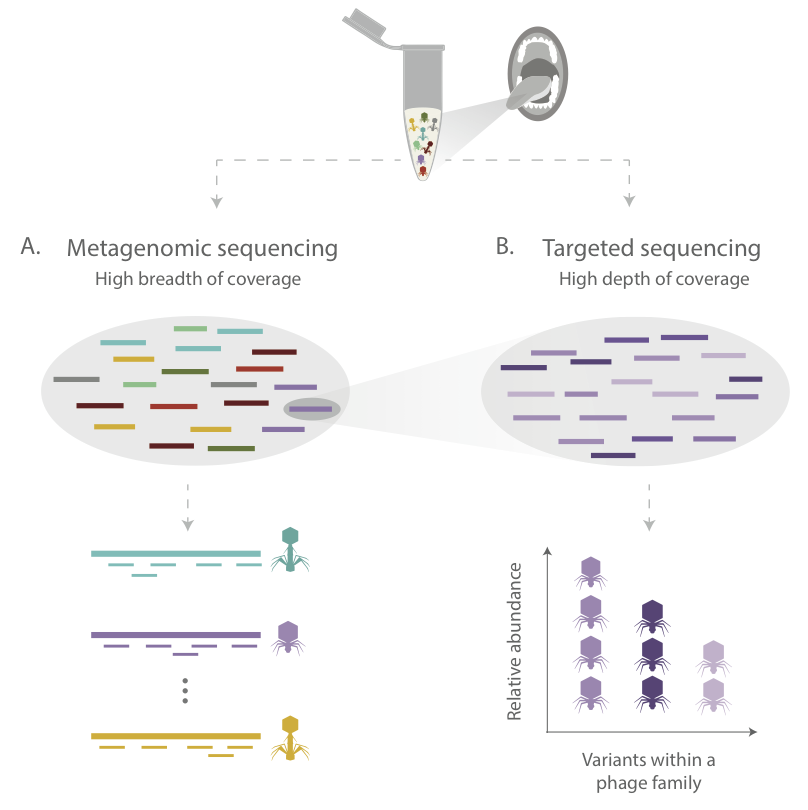


##### **SI Figure 1.** Comparison of A) shotgun metagenomic sequencing and B) targeted sequencing approaches. A) Shotgun metagenomic sequencing offers high breadth of coverage, spanning genomes from many different organisms, however it suffers from low depth of coverage (shown here by the incomplete assembly of phage genomes). B) Targeted sequencing approaches, which use PCR to amplify a specific genomic region, exchange breadth of coverage for depth. Targeted sequencing studies, due to their greater depth of coverage, provide much higher resolution for constructing the communities by equating coverage depth with relative abundance of species or strains.


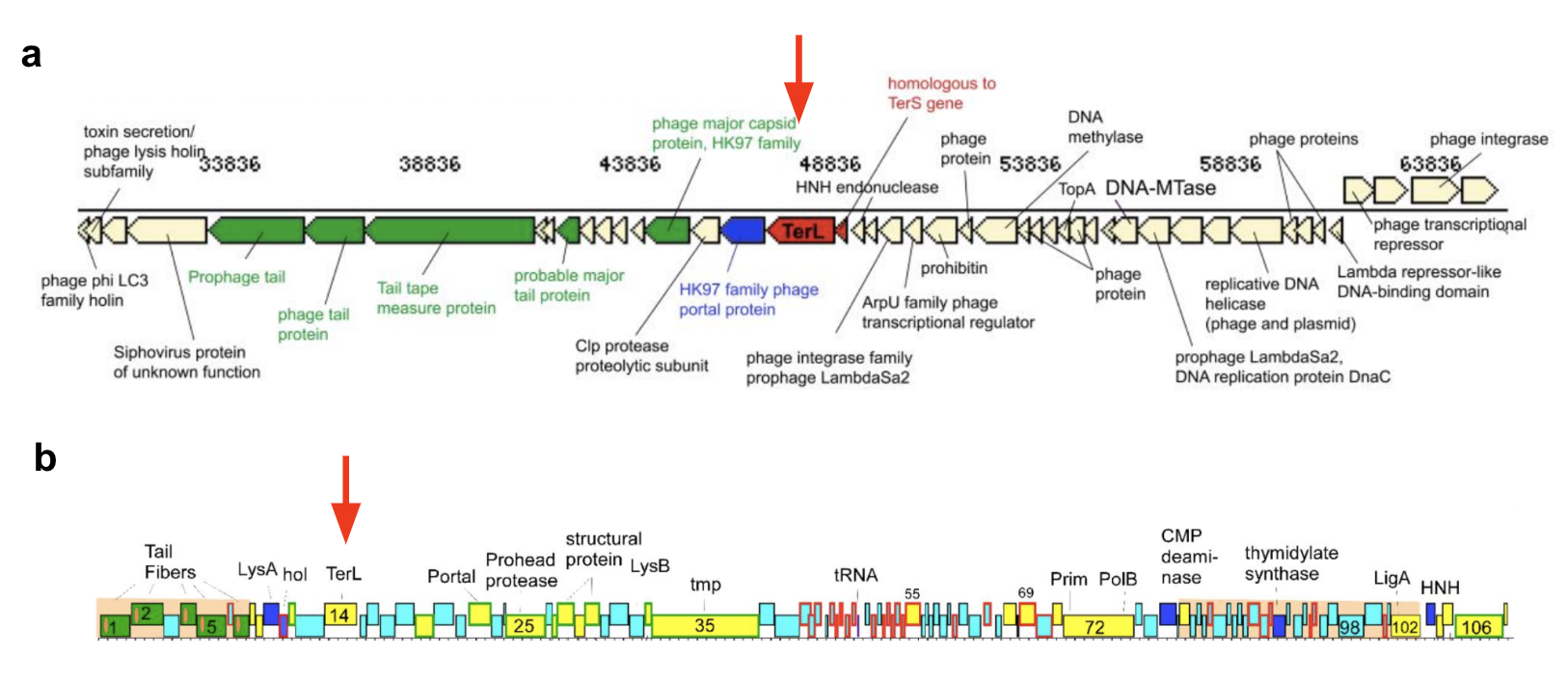
**SI Figure 2.** Genomic positions of HA (a) and HB1 (b) homologous terminases found in publicly available databases. a) *Streptococcus oralis ATCC 49296* phage genome containing HA terminase homolog. Figure adopted from our previous study[^35^](https://www.zotero.org/google-docs/?eeSYZv) b) *Rhodococcus equi ReqiPepy6* phage adopted from Summer *et al*[^64^](https://www.zotero.org/google-docs/?bKiUh2). The red arrows point to the position of the large terminase in these phage genomes.


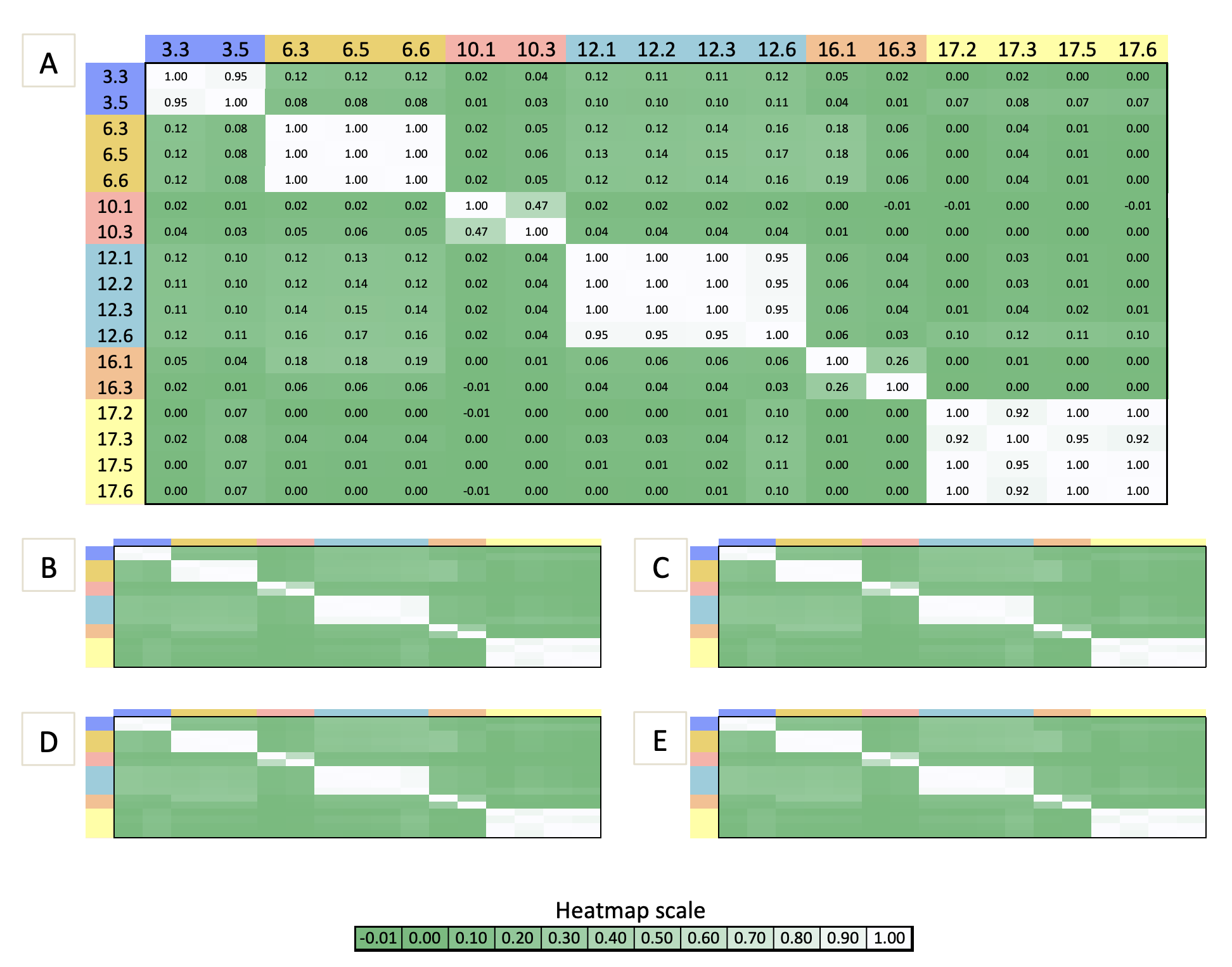
**SI Figure 3.** Pairwise Pearson correlation coefficient values calculated for HB1 phageprints as a function of A) 98%, B) 97%, C) 95%, D) 90%, and E) 80% sequence similarity thresholds for OTU formation. Sample IDs can be decoded as such: subject ID precedes oral site ID. Oral sites 1-6 correspond to tongue dorsum, hard palate, buccal mucosa, ventral tongue, supra-gingiva, and sub-gingiva respectively (e.g. 3.3 corresponds to subject 3 phageprint derived from the buccal mucosa, and 3.5 is subject 3 supra-gingiva phageprint). The number of OTUs generated at 98%, 97%, 95%, 90%, and 80% sequence similarity thresholds are 210, 181, 172, 170, and 80, respectively.


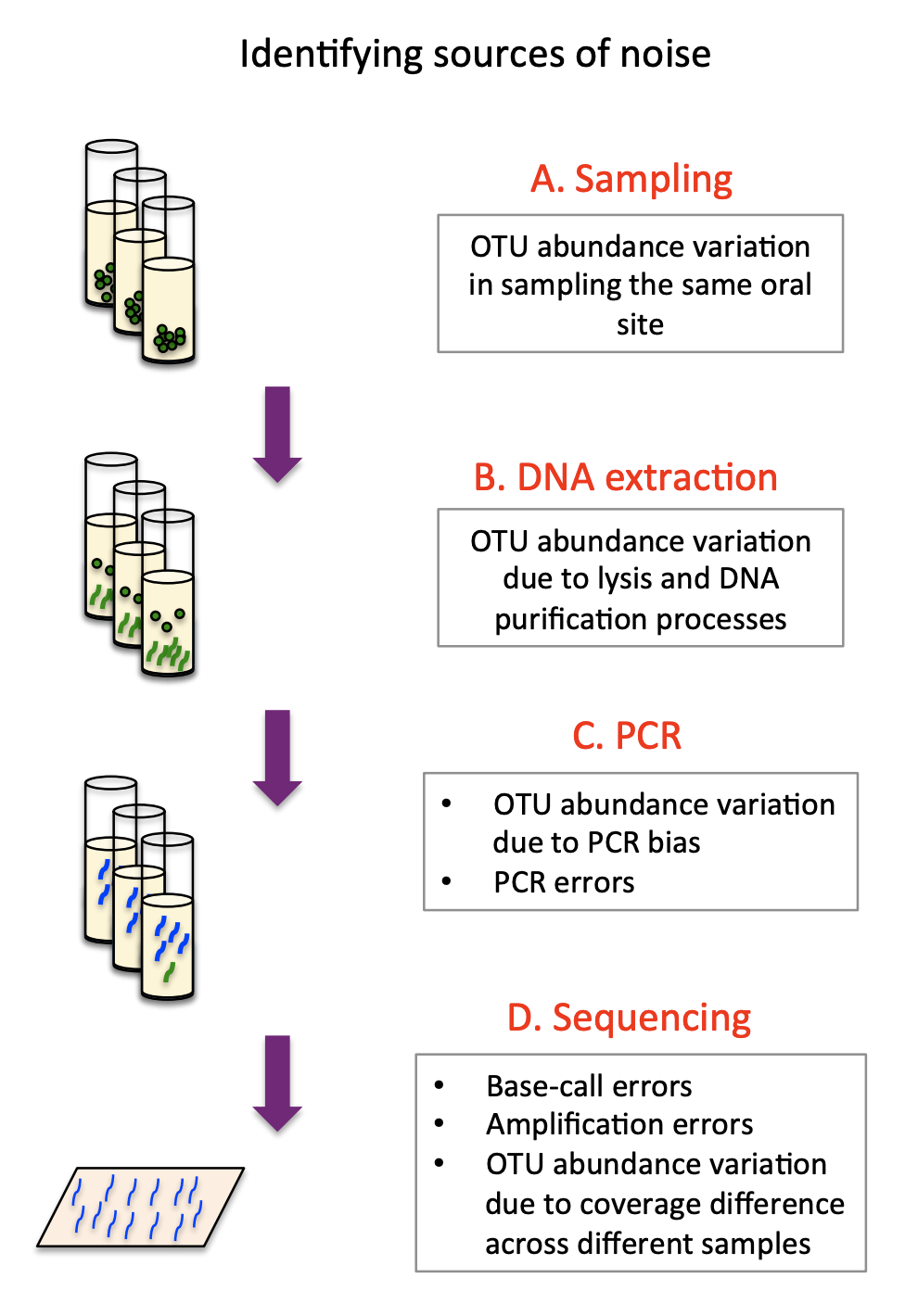


**SI Figure 4.** Sources of error and variation in experimental processes used in this study. A) Sampling of the same oral site in the same individual could result in collection of different microbial communities, which could introduce new OTUs or change relative abundance of existing OTUs. B) DNA extraction is not 100% efficient and the fraction of DNA extracted from an environment could serve as a source of variation across different samples. C) PCR introduces errors that could present themselves as novel OTUs or cause variation in abundance of genuine OTUs. D) Sequencing also introduces errors both at the level of base-calling and bridge amplification.


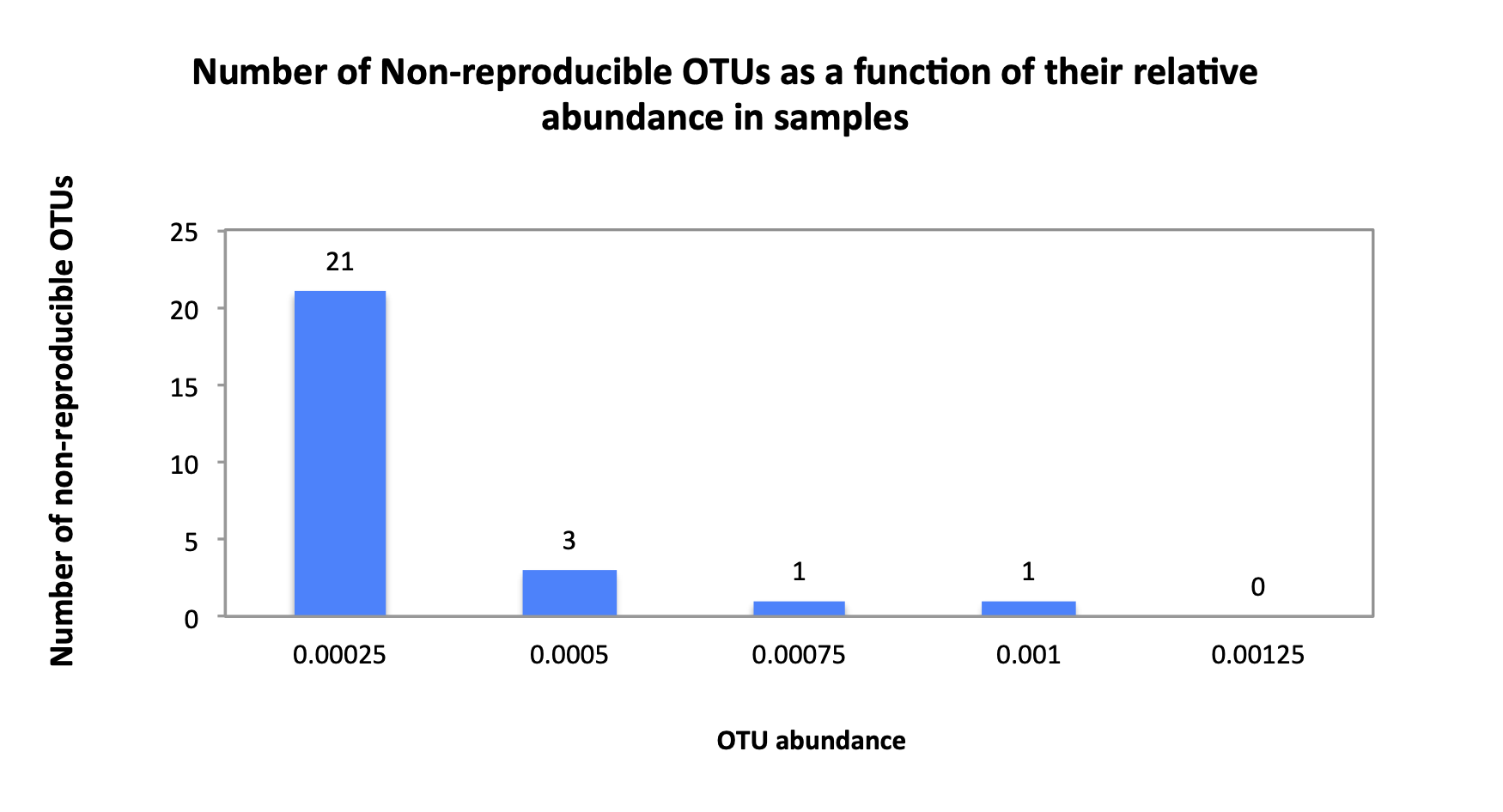


##### **SI Figure 5.** Number of non-reproducible OTUs across three samples obtained from subject 37 tongue dorsum (HB1 terminase family), presented as a function of OTU relative abundance. A total of 30 OTUs appear in one or two samples out of three, and therefore are considered non-reproducible. 21 out of 30 OTUs are defined by a single sequence which translates into 0.00025 relative abundance since samples are rarefied to 4000 sequences. The number of non-reproducible OTUs drops as a function of OTU relative abundance, and all OTUs with more than 4 sequences (0.001 relative abundance) are reproducible across three samples.


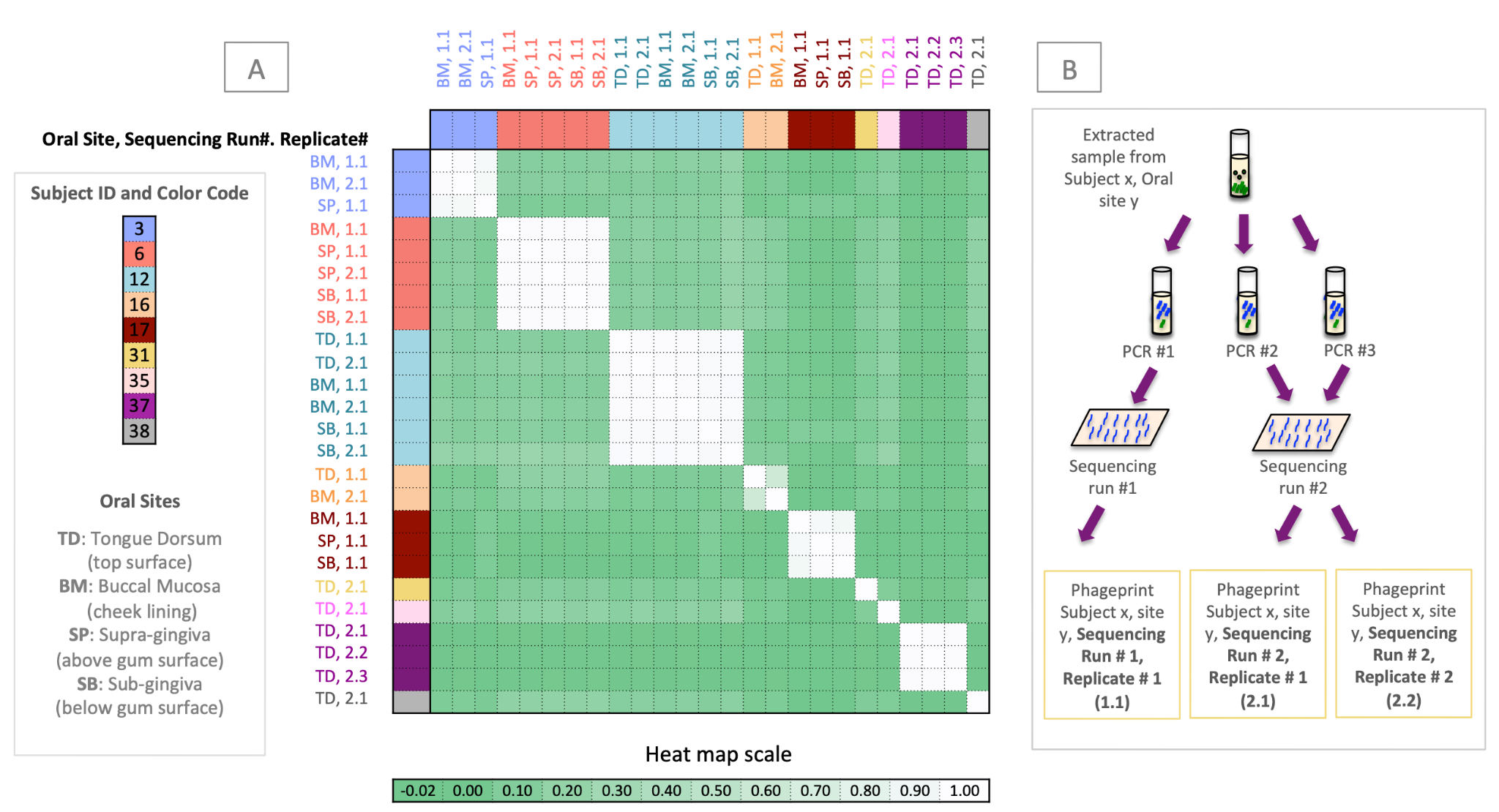


##### **SI Figure 6.** Panel A is the Pearson correlation matrix of all HB1 phageprints. Each phageprint is derived from the analysis of 4000 sequences associated with an individual and a particular oral site. OTUs are defined at 98% sequence similarity and OTUs with less than or equal to 0.001 relative abundance across all phageprints were filtered out. Phageprints are color-coded based on the individual they originate from. Oral sites shown to be positive for the HB1 marker are the tongue dorsum (TD), buccal mucosa (BM), supra-gingiva (SP), and sub-gingiva (SB). Phageprints that were acquired from sequencing run #1, are those marked as replicate #1. Panel B shows that to confirm reproducibility of phageprints, a second set of PCR was performed on previously extracted DNA from all samples included in sequencing run #1 and those PCR products were included in sequencing run #2. Phageprints derived from the second sequencing run are marked as replicate #2.


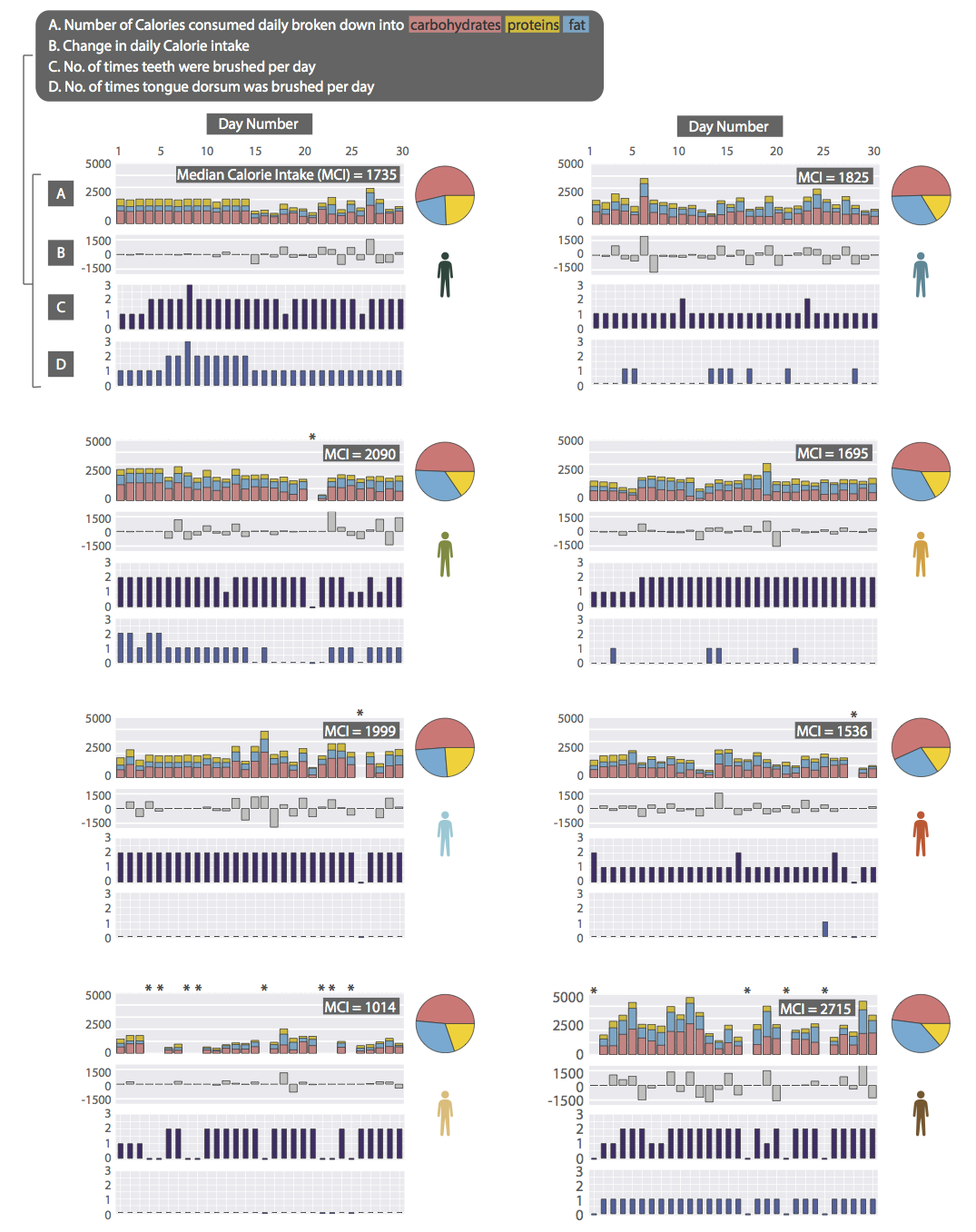


##### **SI Figure 7.** Subject daily metadata for the temporal cohort. Top panel for each subject represents the Caloric intake from fats, carbohydrates, and protein. Mean Caloric Intake or MCI reports the Caloric intake averaged over the sampling period (the x-axis for all plots is number of days). Pie charts demonstrate the diet over sampling days based on median fat, carbohydrate and protein consumption. The second panel depicts the change in Calorie intake from the previous day. The third and fourth panel correspond to the number of times that the subject brushed his or her teeth and tongue, respectively, during the 24 hour sampling interval. We have used an asterisk to denote days for which we did not receive data from the subject, and to distinguish them from zero values in the third and fourth panel, they have been given “-.1” value. Two subjects did not report dietary information so they are not included in this figure.


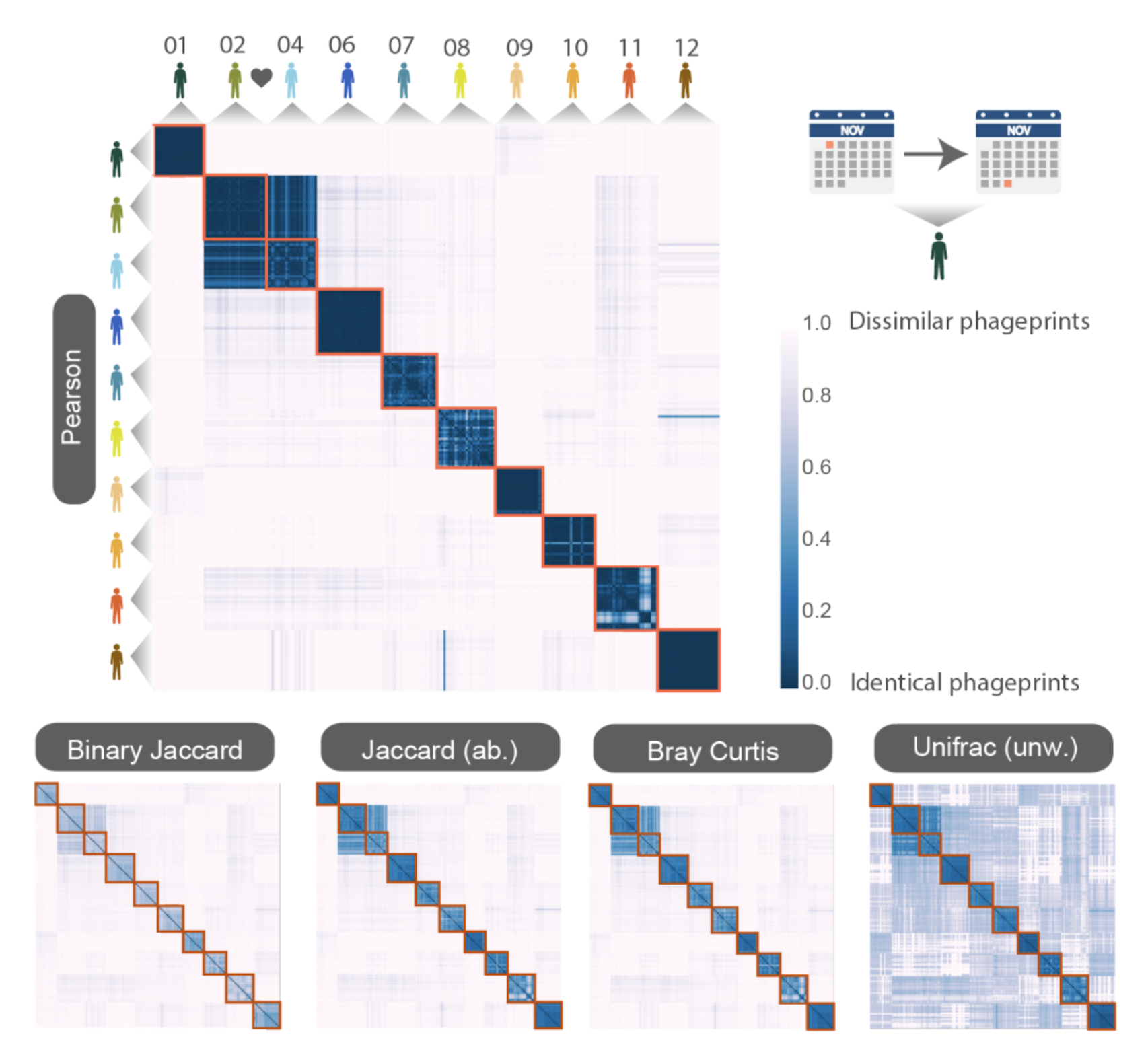


##### **SI Figure 8.** HB1 phageprints temporal dynamics depicted here by pairwise distance metrics: A) Pearson, B) Binary Jaccard, Abundance Jaccard, Bray Curtis and unweighted Unifrac. The heatmap scale applies to all heatmaps shown. Subjects 02 and 04 are partners. Samples from each subject are chronologically ordered.


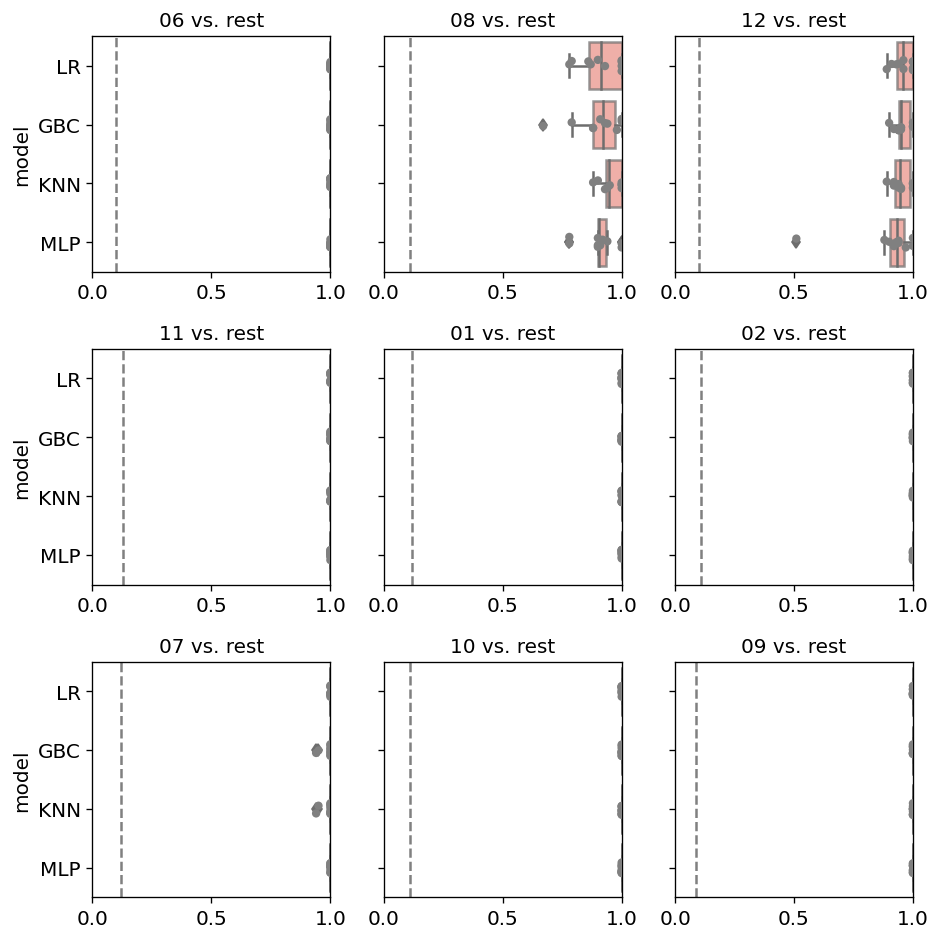
**SI Figure 9.** Four types of machine learning models are built to classify one individual’s phageprints from the rest (HA phageprints). For each model type, 10 independent models based on 10 different train/test splits are built, and boxplots of the Area Under the Precision Recall Curve (AUPR) is reported in this table. The null value (i.e. prevalence of the positive class) is shown as a dashed line.


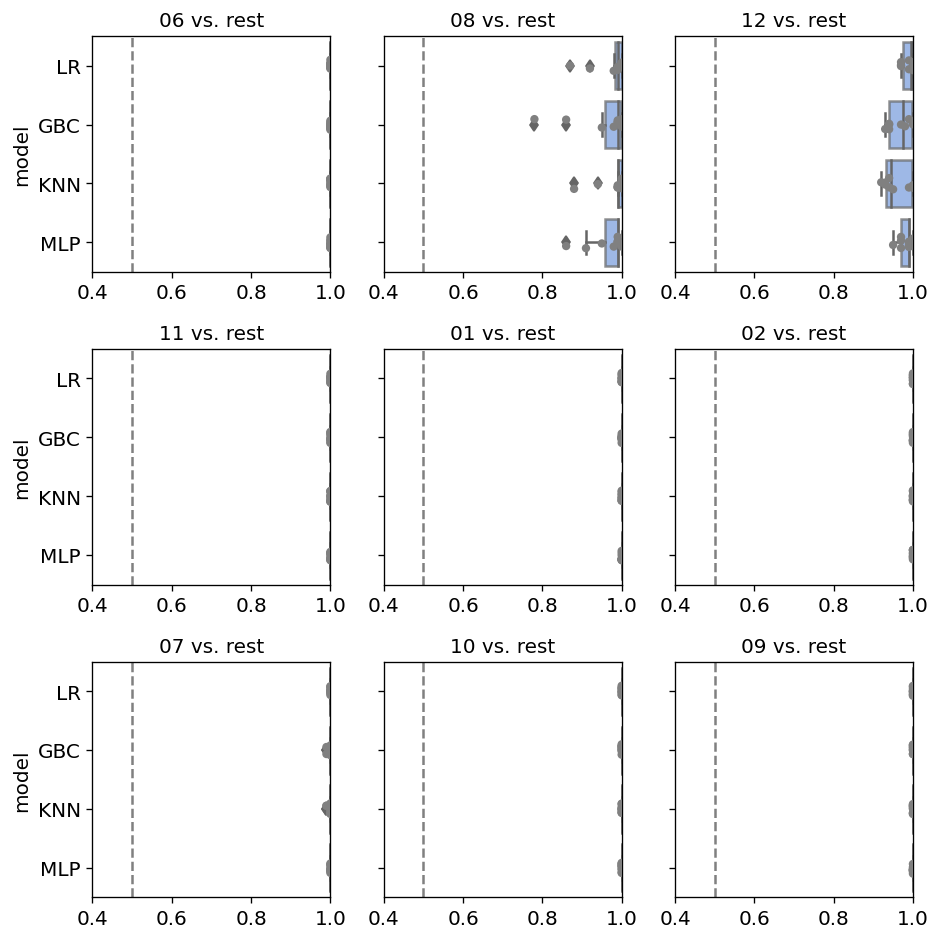


##### **SI Figure 10.** Four types of machine learning models are built to classify one individual’s phageprints from the rest (HA phageprints). For each model type, 10 independent models based on 10 different train/test splits are built, and boxplots of the Area Under the Receiver Operator Curve (AUROC) is reported in this table. The null value (0.5) is shown as a dashed line.


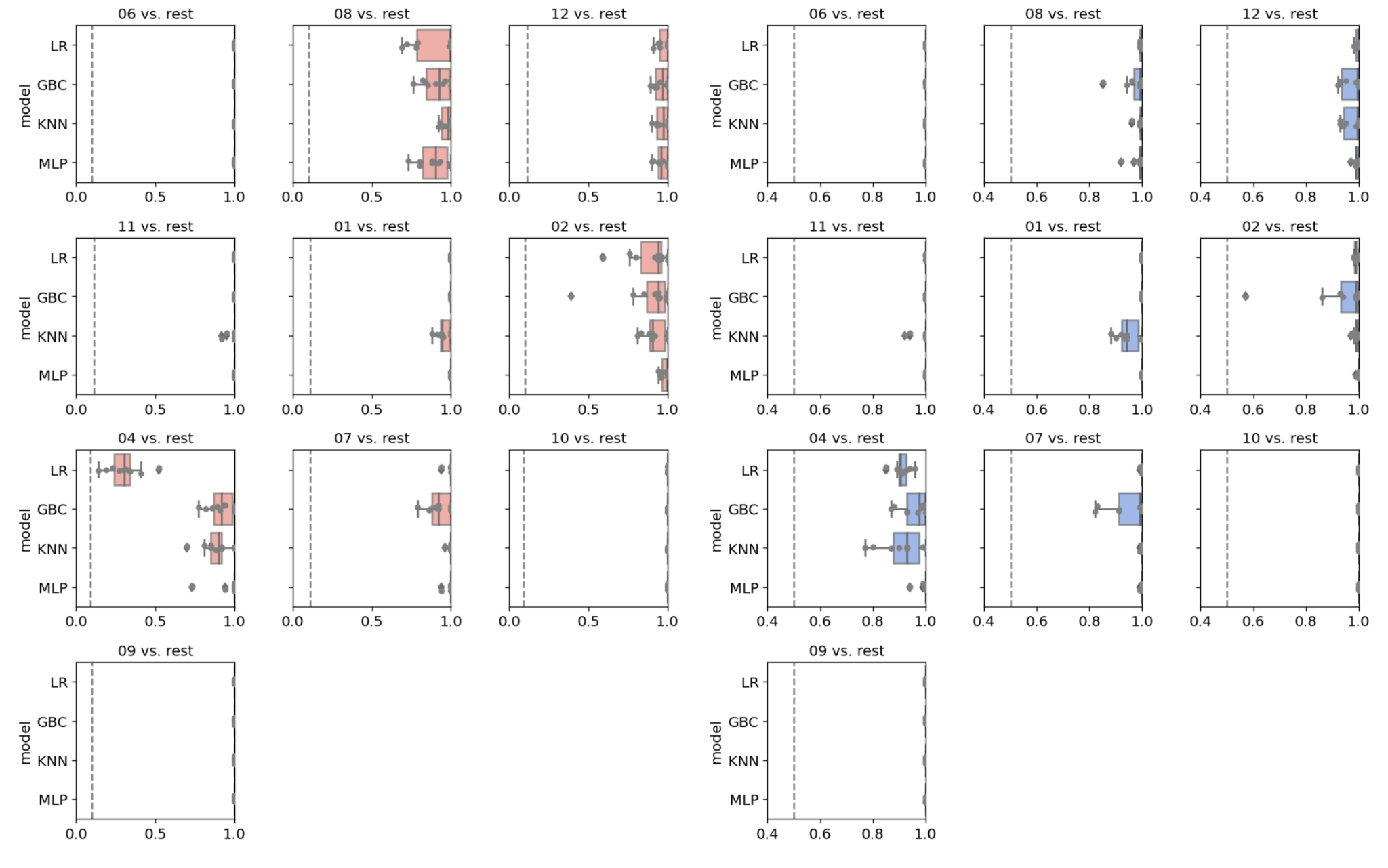
**SI Figure 11.** Machine learning one-versus-rest models built to distinguish between one person’s HB1 phageprints from the rest, including subject 4 in the cohort. Salmon and blue panels represent boxplots of the Area Under the Precision Recall (AUPR) Curve and the Area Under the Receiver Operator Curve (AUROC) values, respectively. The null values are shown as dashed lines, which for AUROC is equal to 0.5 and for AUPR is equal to the prevalence of the positive class. The four model types shown on the y axes are Logistic Regression (LR), Gradient Boosting Classifier (GBC), K-Nearest Neighbor (KNN), and Multi-Layer Perceptron (MLP). For each model type 10 models are built based on 10 different splits of the data into training and testing portions. Subject IDs are shown at the top of each panel, such that “06 vs. rest” for instance, corresponds to model performances on test data distinguishing subject 6 phageprints from all other subjects’ phageprints.


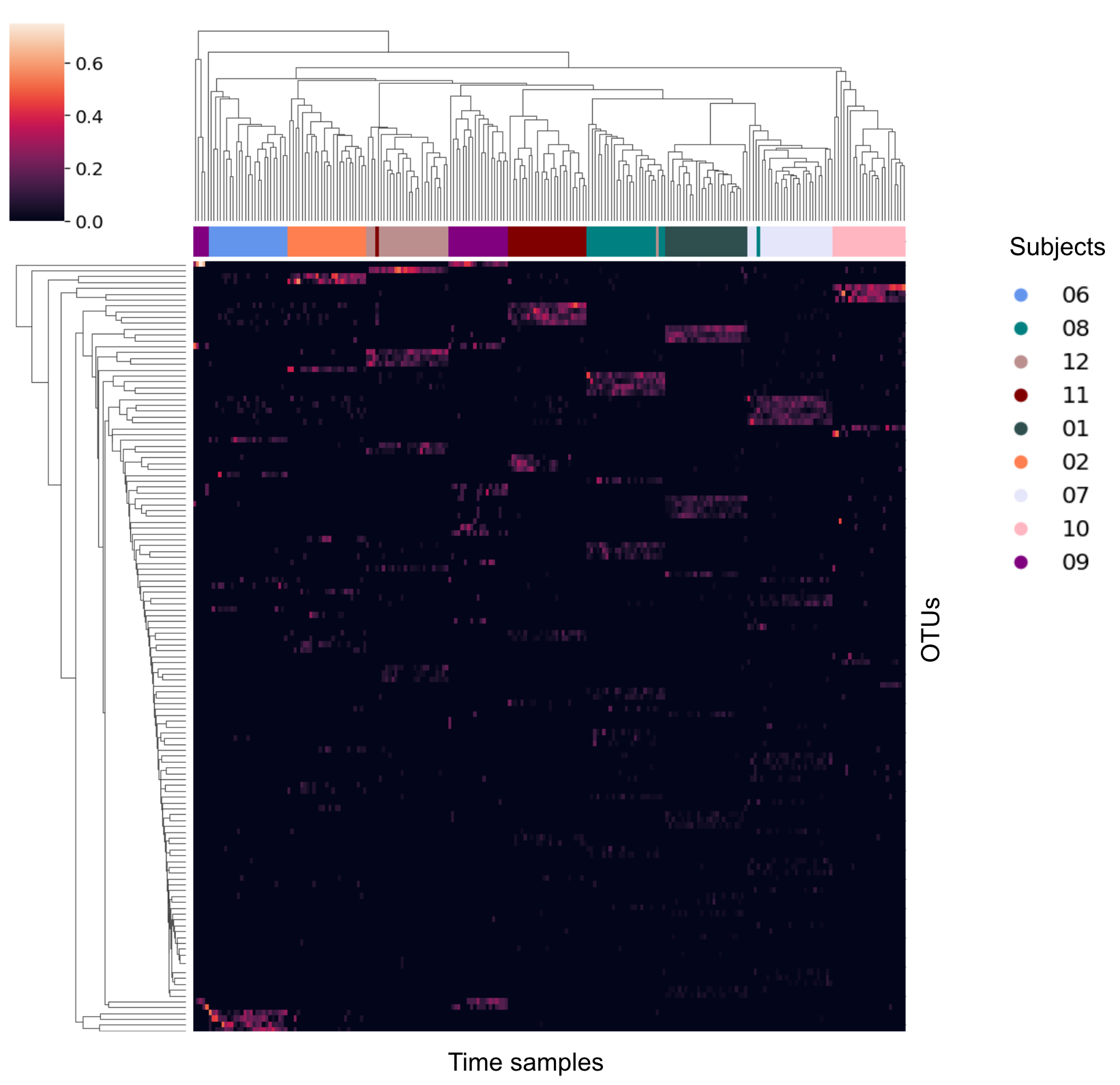
**SI Figure 12.** Hierarchical clustering of samples from different subjects based on 226 OTUs which were randomly subsampled from a reduced OTU table. The reduced OTU table did not contain the top 10 most abundant OTUs of each sample, which collectively resulted in the removal of 577 most abundant OTUs. Time samples from each subject are color-coded, and the relative abundance of each OTU is shown in this clustermap.


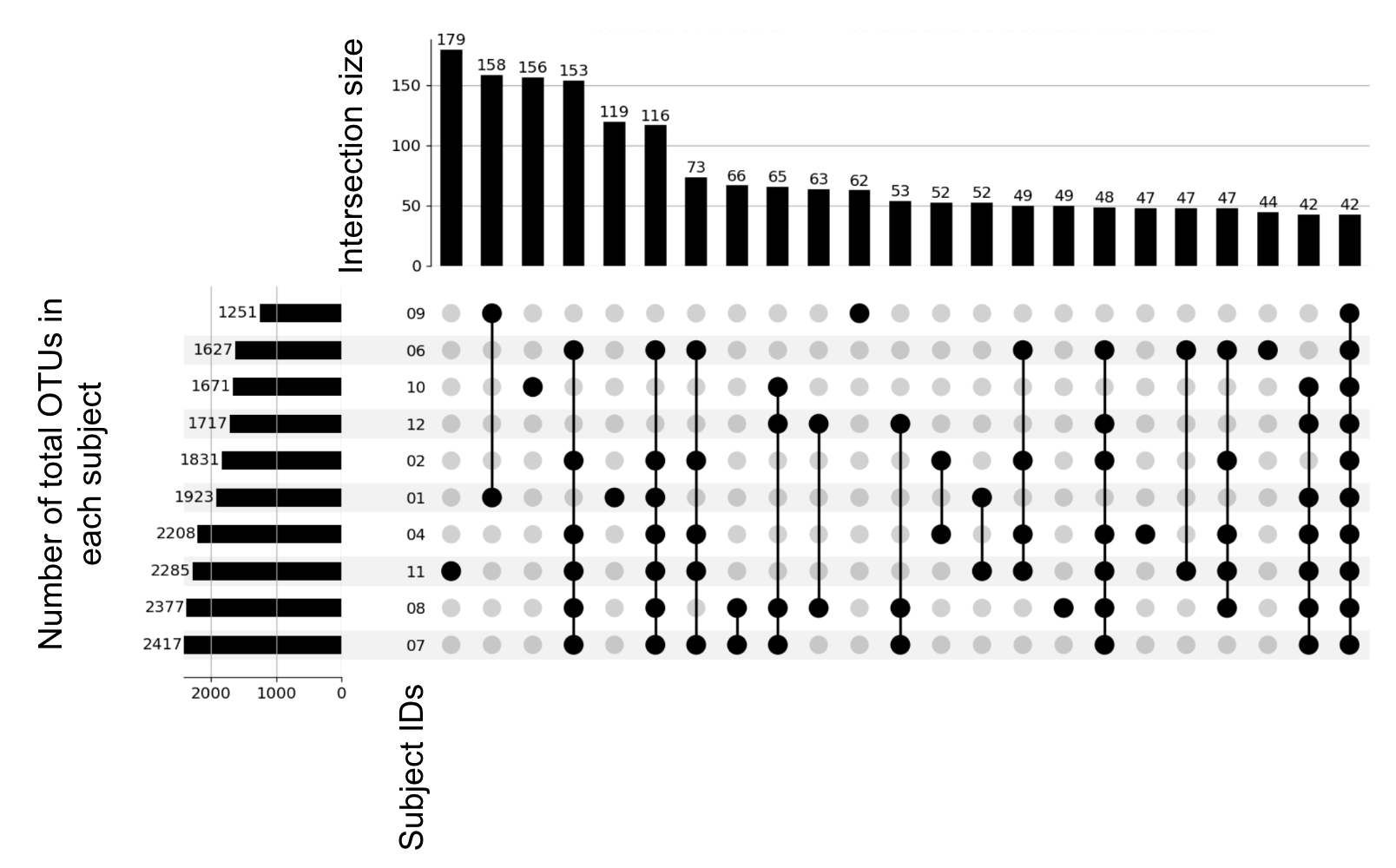


##### **SI Figure 13.** An UpSet plot showing the number of OTUs unique to each subject as well as the number of OTUs shared between different subjects. Connected dots represent sharing of OTUs between one or more subjects, whereas lone dots are sets of OTUs unique to an individual. Sets are ordered based on size (vertical bars). The counts of total OTUs in a subject are shown as horizontal bars for each subject. For visual clarity, sets shown have a minimum size of 40 OTUs.


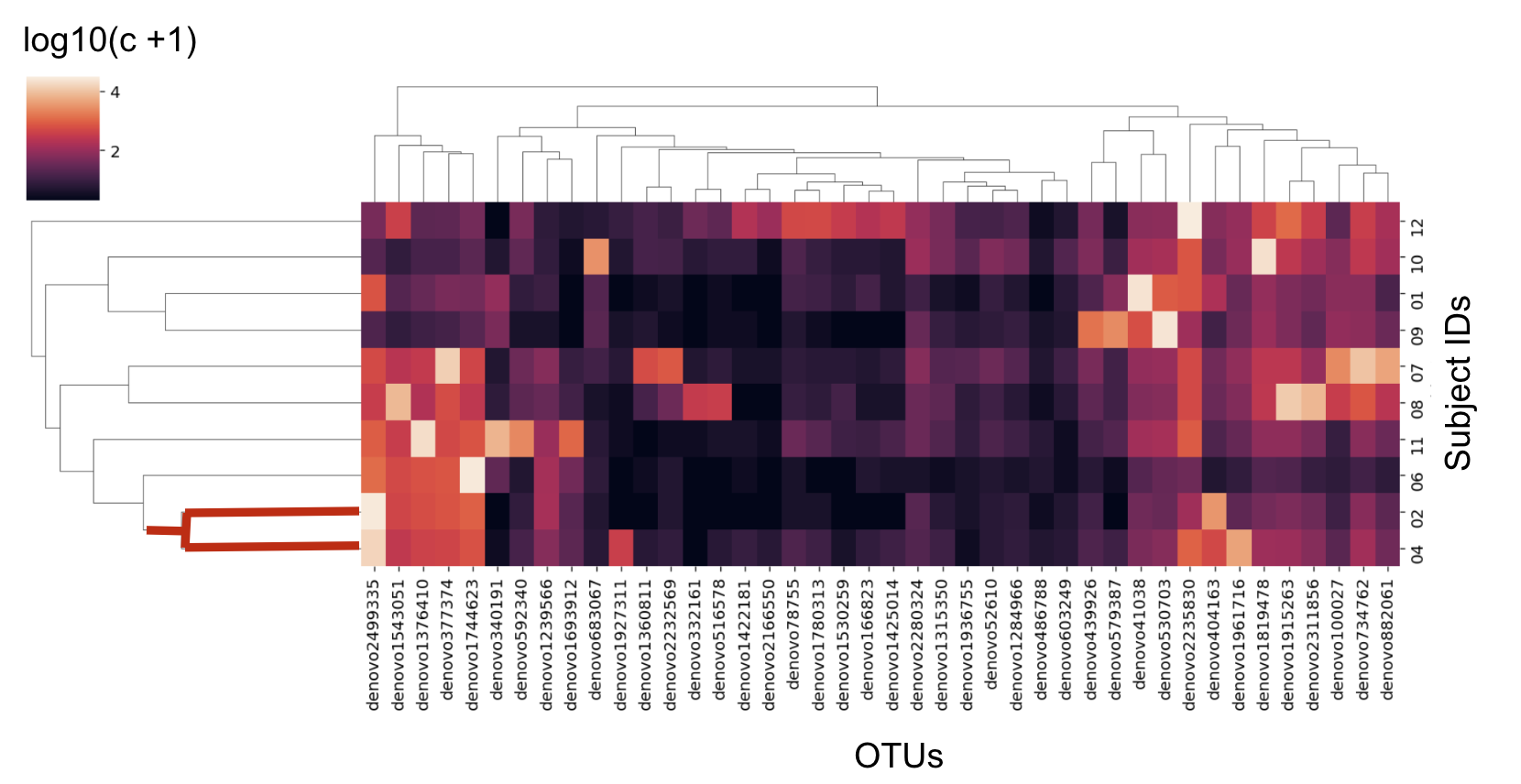


##### **SI Figure 14.** Hierarchical clustering of generalist OTUs- those OTUs found across all subjects within the temporal study- based on their abundance. Counts, c, are based on the abundance of each OTU summed across all time points within a subject. Log10 of counts + 1 is shown in the clustermap. Samples from subjects 2 and 4 who are partners cluster most closely to each other than to other samples.

####
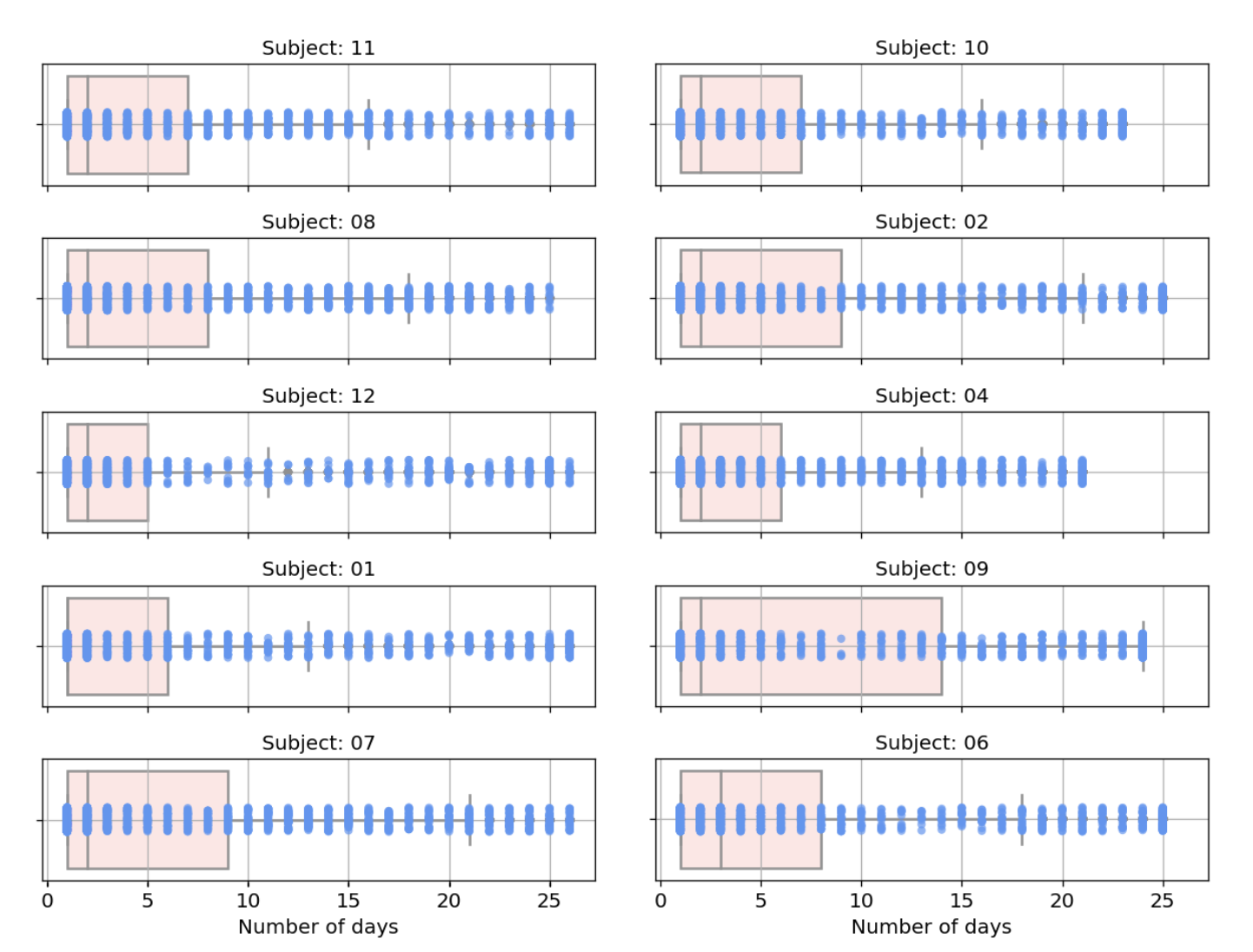


##### **SI Figure 15.** Boxplots (shown in pink) and swarmplots (blue) of the total number of days that each OTU was observed within an individual. Blue dots correspond to individual OTUs.


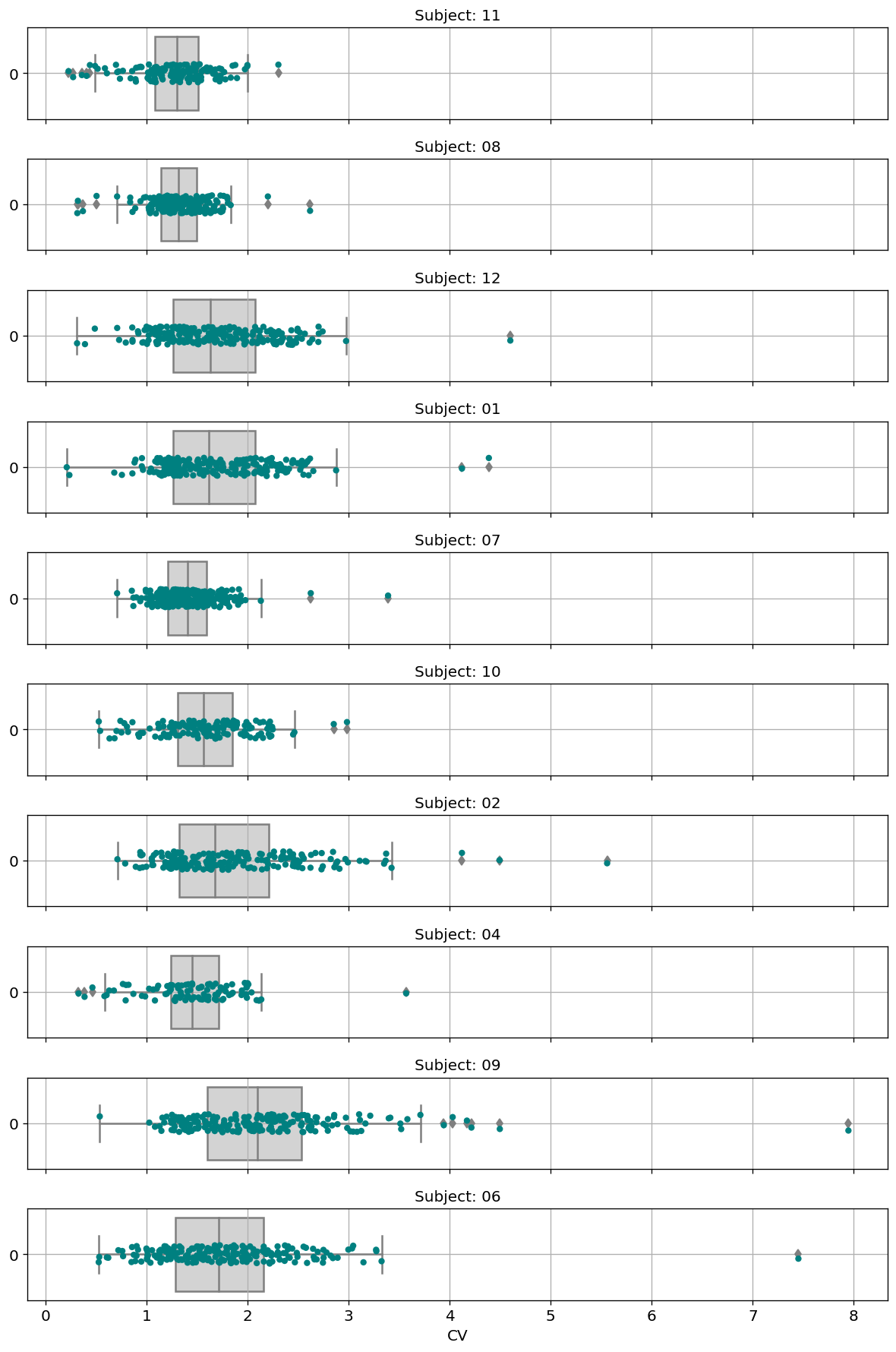


##### **SI Figure 16.** Boxplots and swarmplots showing within each subject the coefficient of variation of persistent OTUs across the sampling period. Persistent OTUs are those that appear in at least 20 days.

####
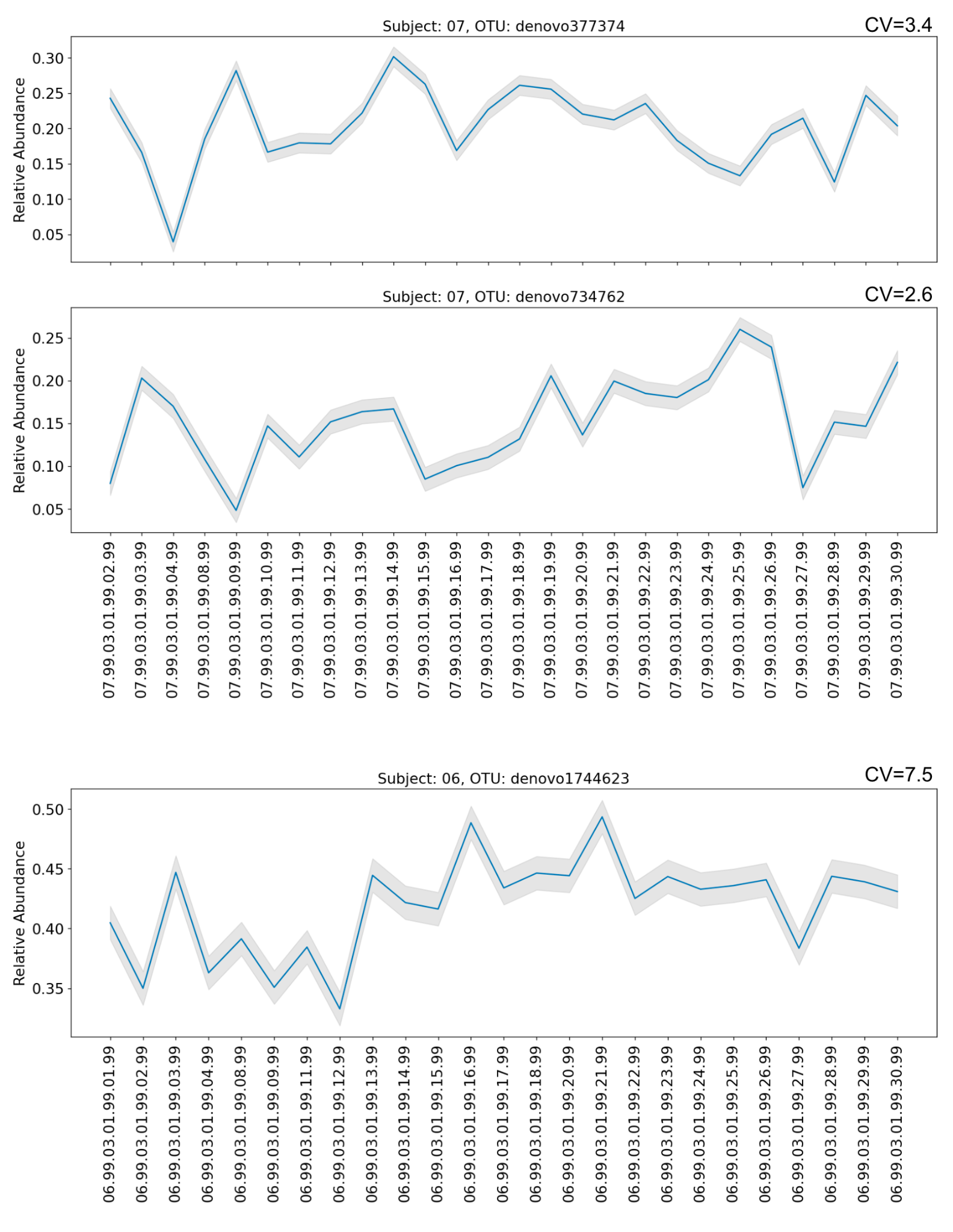


##### **SI Figure 17.** Representative plots of relative abundance as a function of time for outlier OTUs with high coefficient of variation (shown at the top right corners of each plot) in subjects 7 and 6. Gray regions correspond to experimental margin of error. They represent 2 standard deviations above and below the mean. Because only one sample per time point is taken per individual, we conservatively use the maximum standard deviation observed in our noise measurement experiments.


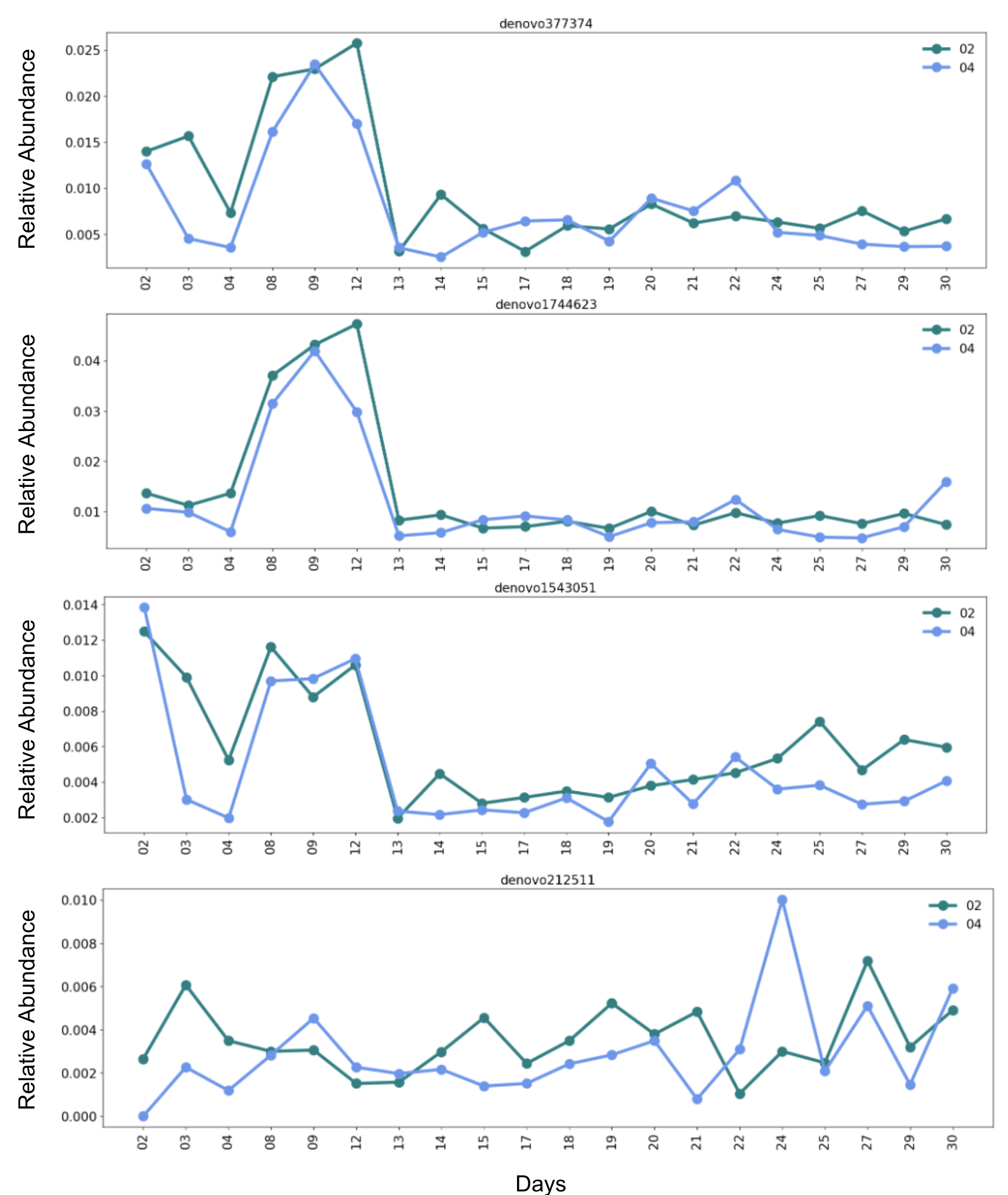


##### **SI Figure 18.** Four OTUs relative abundances shown as a function of time in subjects 2 and 4, who are partners. Only sampling days (x axis) that were made available by both partners are shown for easier comparison.


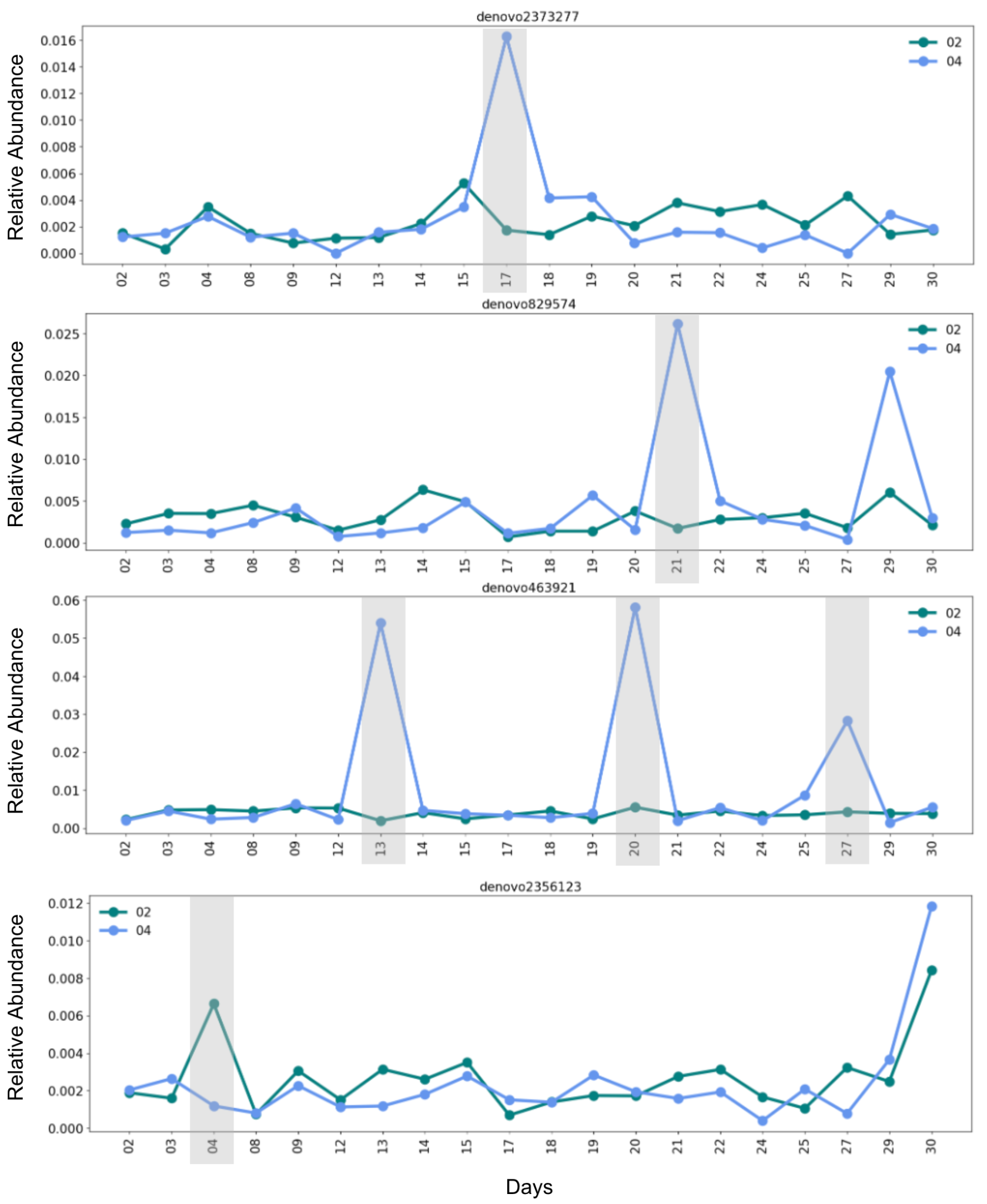
**SI Figure 19.** Four OTUs relative abundances shown as a function of time in subjects 2 and 4, who are partners. Grey regions are drawn around time points where there is a several fold change in the relative abundance of a given OTU in one partner but not the other. Only sampling days that were not missed by both partners are shown for easier comparison.


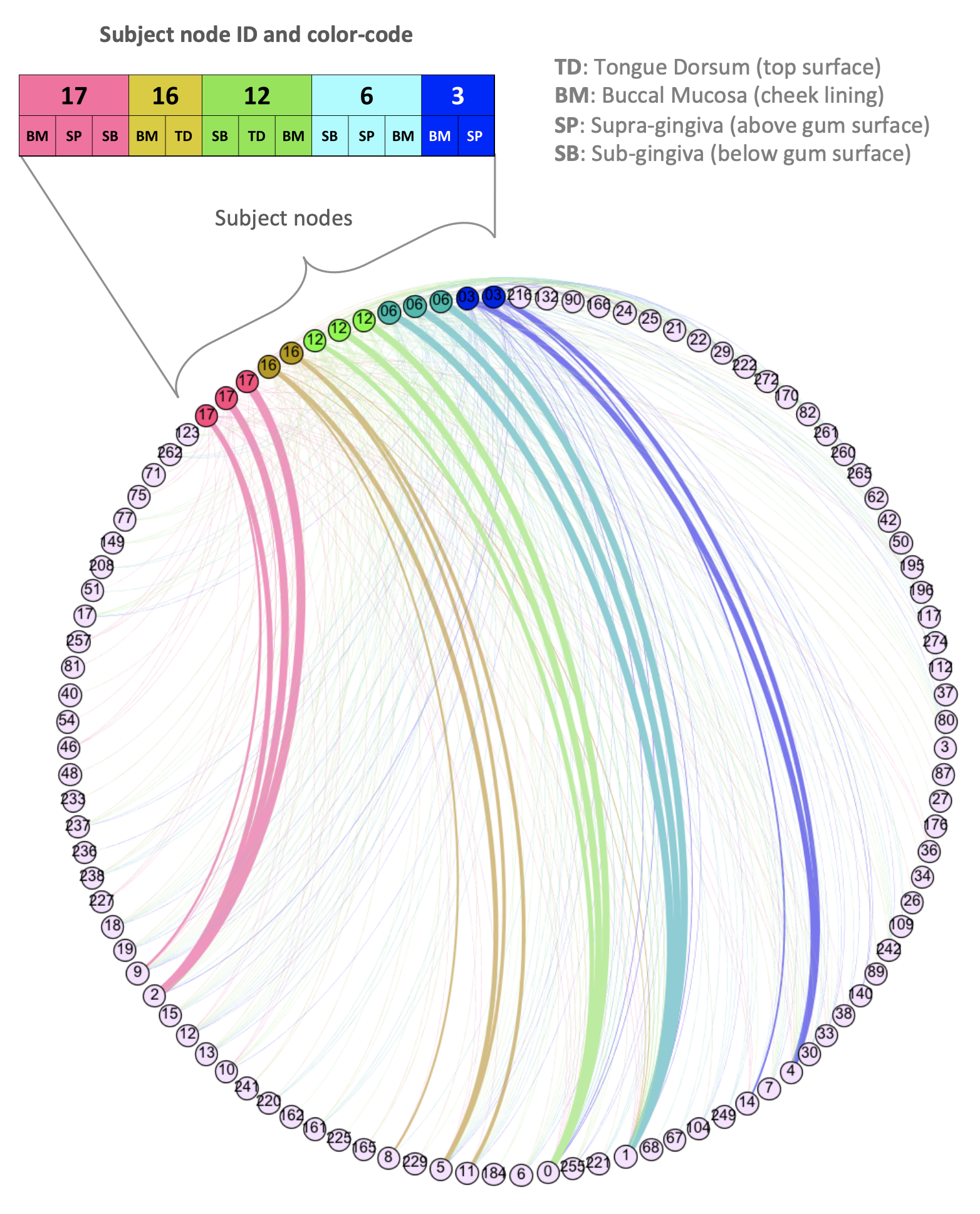


##### **SI Figure 20.** HB1 phage family network. Purple nodes are the OTU nodes and all other nodes represent samples. Sample nodes and edges are color-coded based on the individual they originate from. The oral site associated with each sample is abbreviated next to the sample’s node. Each edge connects an OTU to a sample it exists in, and the edge weight is proportional to the relative abundance of the OTU in that sample. Node IDs are displayed. For OTU nodes, the node ID is the OTU ID. For sample nodes, the nodes IDs are simply the subjects’ IDs.


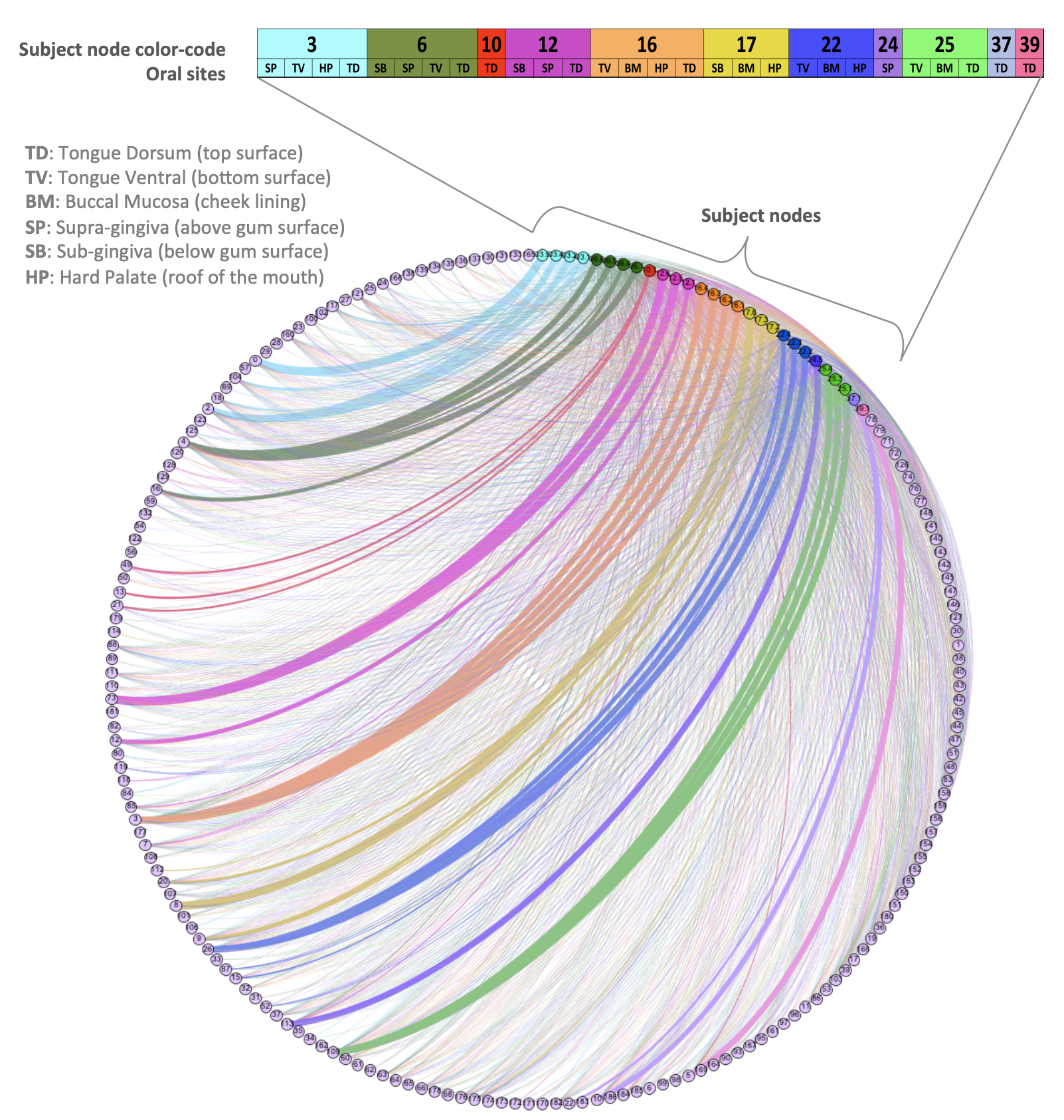


##### **SI Figure 21.** HA phage-host network. Purple nodes are the OTU nodes and all other nodes represent samples. Sample nodes and edges are color-coded based on the individual they originate from. Subject node color code, ID, and the oral sites are displayed above sample nodes. Each edge connects an OTU to a sample it exists in, and the edge weight is proportional to the relative abundance of the OTU in that sample. Node IDs are displayed. For OTU nodes, the node ID is the OTU ID. For sample nodes, the nodes IDs are simply the subjects’ IDs.


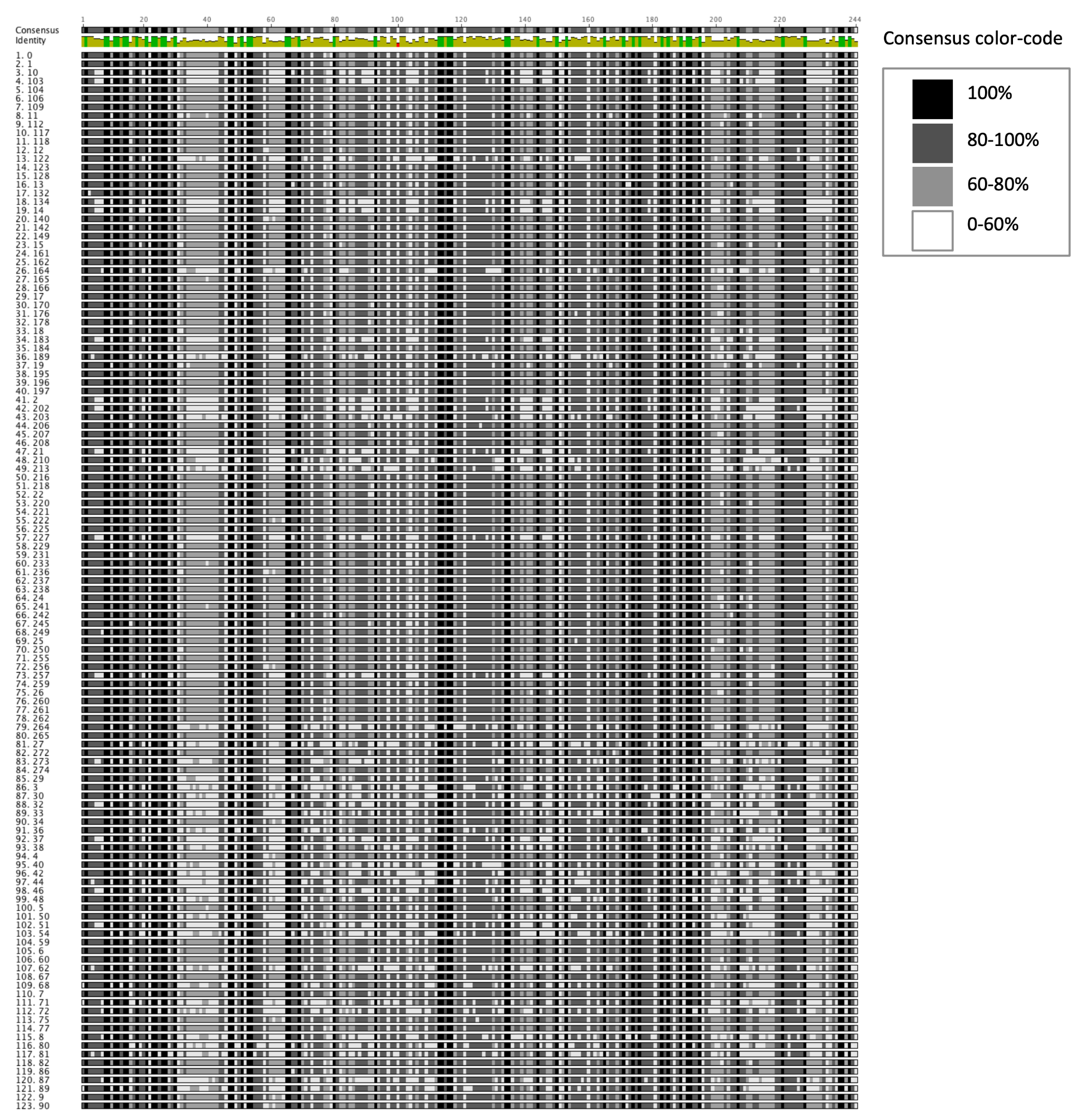


##### **SI Figure 22.** The nucleotide alignment of HB1 phage family OTU representative sequences. Sequences were aligned using Geneious software. No gaps were introduced. Each base is color-coded according to its relative abundance within a column in the alignment. Conserved bases are black and highly variable sites are shown in white.

#### **SI Tables**

##### **SI Table 1.** HA and HB1 primer sequences. Please refer to our earlier manuscript for more information about primer design[^35^](https://www.zotero.org/google-docs/?JAo8fK).

| **Terminase family** | **Primer name** | **Primer** |
| --- | --- | --- |
| HA | HA.F1 (forward) | 5'-CGTGATGGCTGYCTWGARTTYGAYGA-3' |
|  | HA.R1 (reverse) | 5'-CGTAAGGAGTGCTYTCRTCCARCATIGG-3' |
| HB1 | HB1.F2 (forward) | 5'-CCGATCTGTCICARGGIGAYGA-3' |
|  | HB1.R1 (reverse) | 5'-GTTACGAACTCTTTGGCRTTRTAIGGRTC-3' |

##### **SI Table 2.** Four types of machine learning models are built to classify one individual’s phageprints from the rest (for HB1 terminase family). For each model type, 10 independent models based on 10 different train/test splits are built, and the 95% confidence interval for the Area Under the Precision Recall (AUPR) curve is reported in this table. For instance, column one shows the performance of all model types for detecting subject 6’s phageprint from the rest.


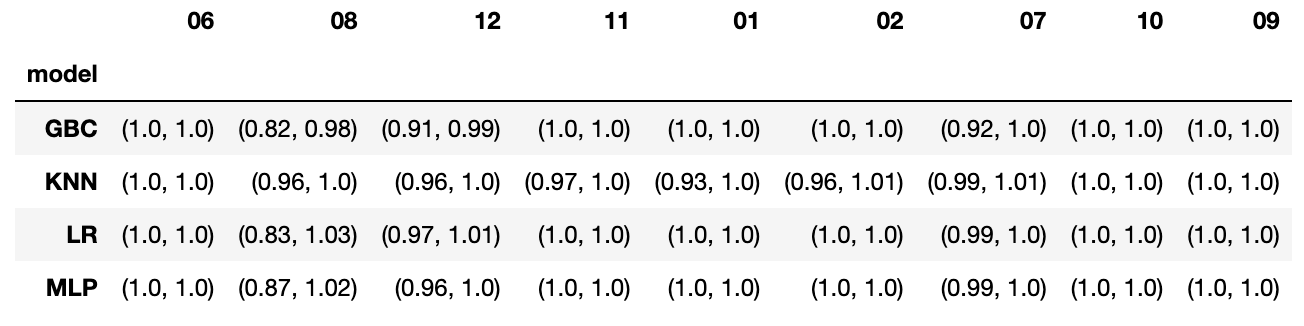


##### **SI Table 3.** Four types of machine learning models are built to classify one individual’s phageprints from the rest (for HB1 terminase family). For each model type, 10 independent models based on 10 different train/test splits are built, and the 95% confidence interval for the Area Under the Receiver Operator curve (AUROC) is reported in this table. For instance, column one shows the performance of all model types for detecting subject 6’s phageprint from the rest.


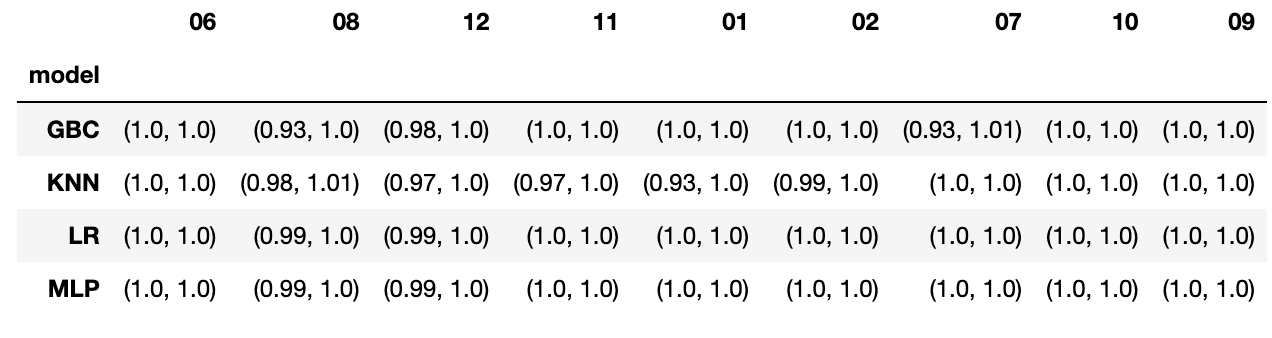


**SI Table 4.** Four types of machine learning models are built to classify one individual’s phageprints from the rest (for HA terminase family). For each model type, 10 independent models based on 10 different train/test splits are built, and the 95% confidence interval for the Area Under the Receiver Operator curve is reported in this table.
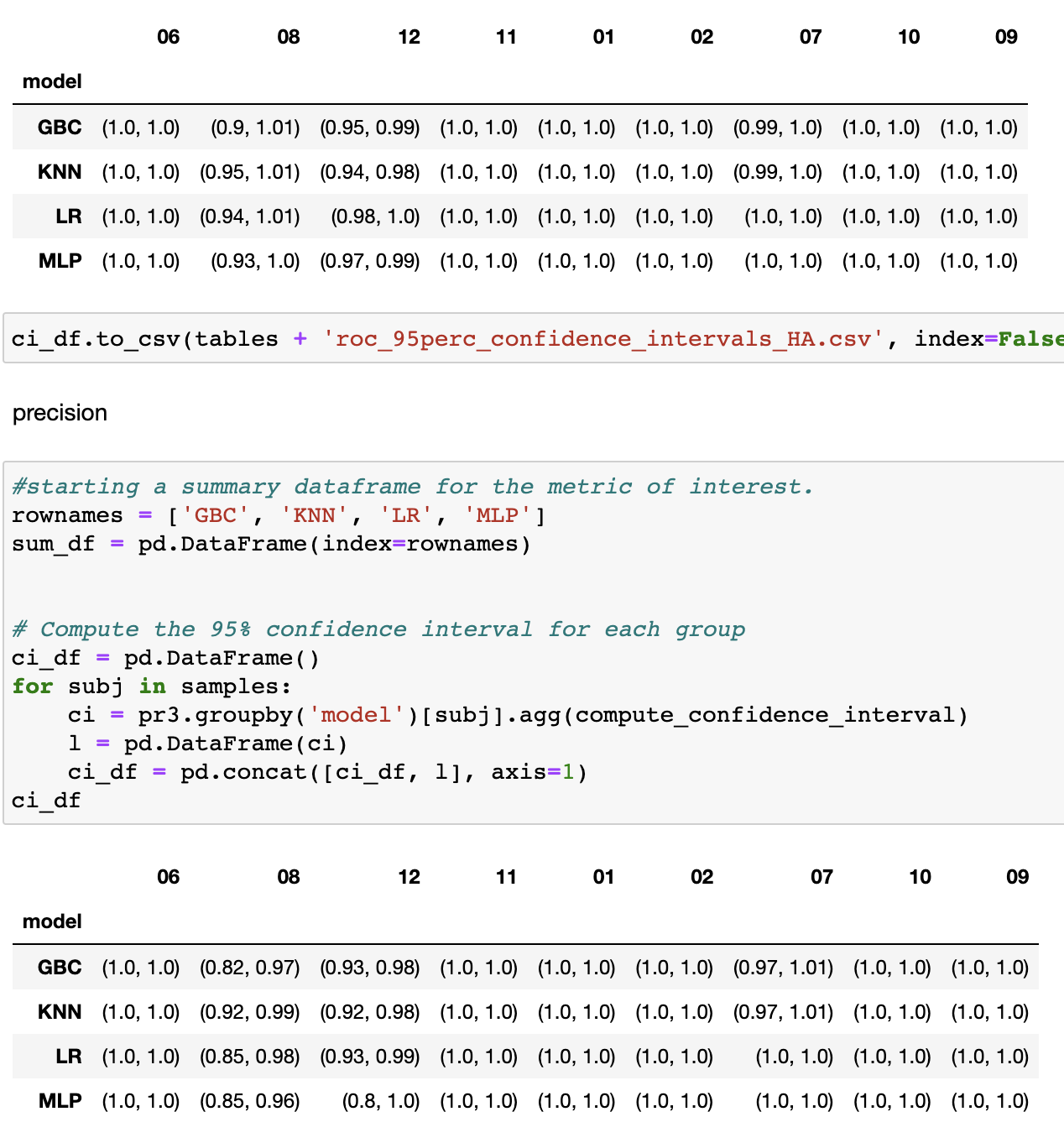


##### **SI Table 5.** Four types of machine learning models are built to classify one individual’s phageprints from the rest (for HA terminase family). For each model type, 10 independent models based on 10 different train/test splits are built, and the 95% confidence interval for the Area Under the Precision Recall curve is reported in this table.


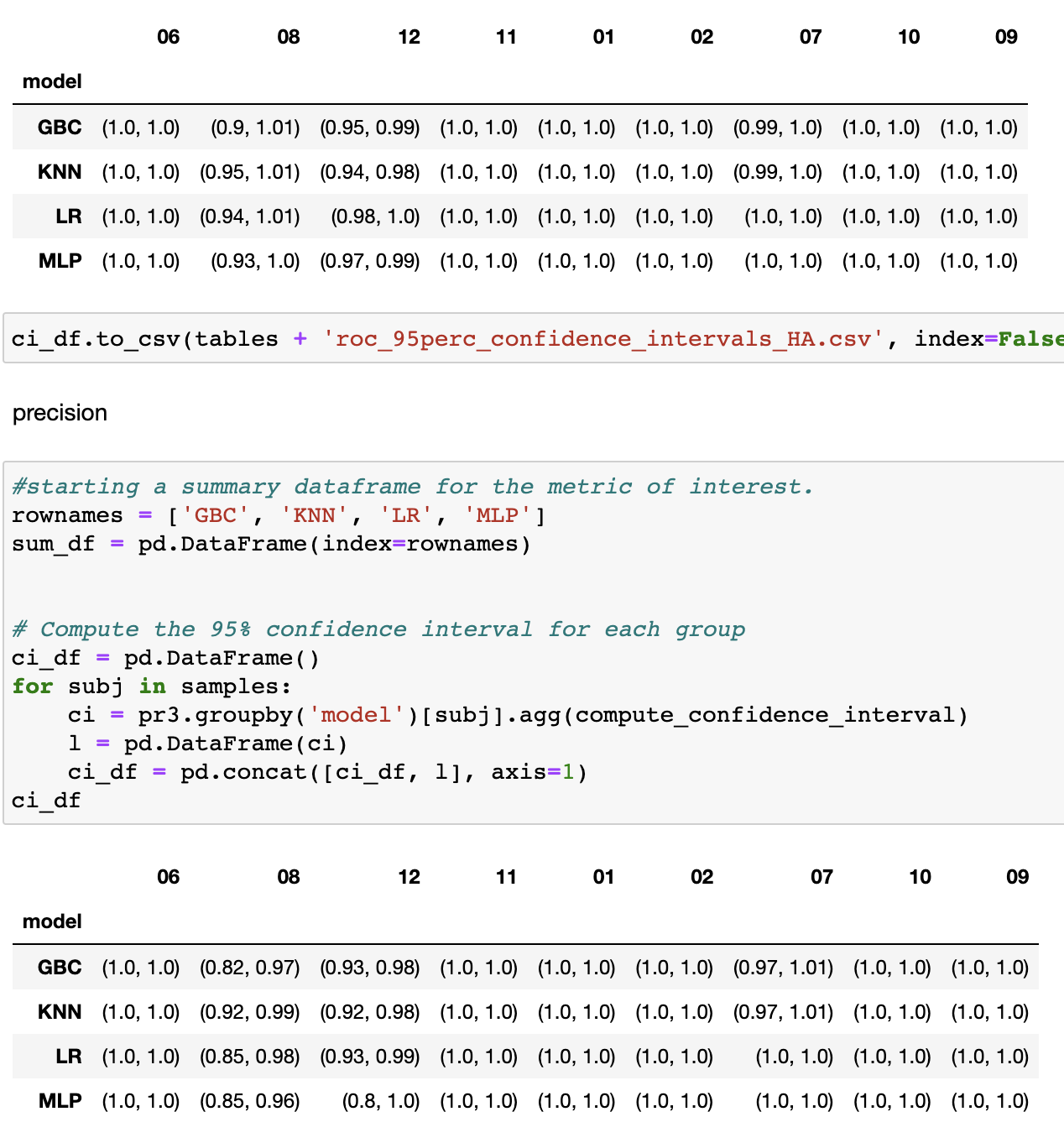


**SI Table 6.** Logistic regression one-versus-rest models built to classify one individual’s HB1 phageprints after ~600 dominant OTUs have been removed from the dataset. For each model type, 10 independent models based on 10 different train/test splits are built, and the 95% confidence intervals, mean and standard deviation for the Area Under the Receiver Operator Curve (AUROC) is reported in this table.


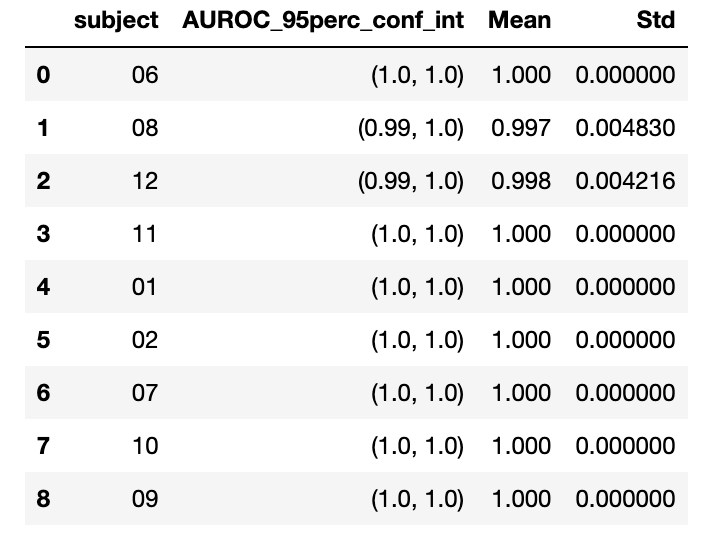


##### **SI Table 7.** Logistic regression one-versus-rest models built to classify one individual’s HB1 phageprints after ~600 dominant OTUs have been removed from the dataset. For each model type, 10 independent models based on 10 different train/test splits are built, and the 95% confidence intervals, mean and standard deviation for the Area Under the Precision Recall curve (AUPR) is reported in this table.

##### **
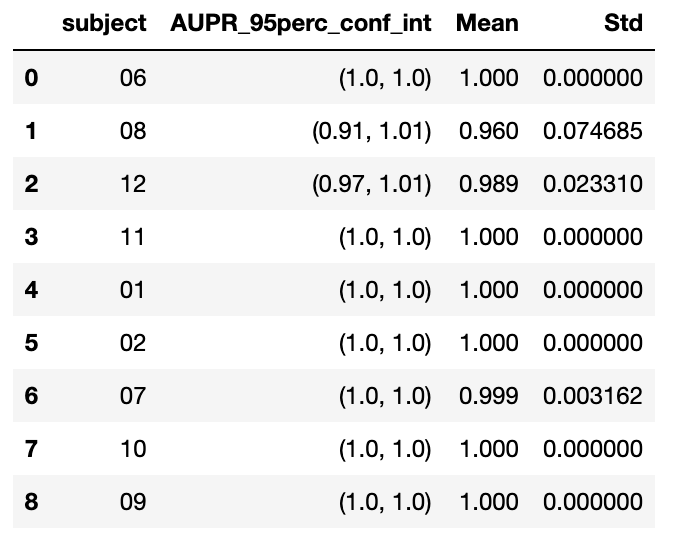
**

##### **SI Table 8.** Logistic regression one-versus-rest models built to classify one individual’s HB1 phageprints after ~600 dominant OTUs were removed and the resulting OTU table was subsampled to contain only 2% of the total OTUs (~200). For each model type, 10 independent models based on 10 different train/test splits are built, and the 95% confidence intervals, mean and standard deviation for Area Under the Receiver Operator Curve (AUROC) is reported in this table.

**
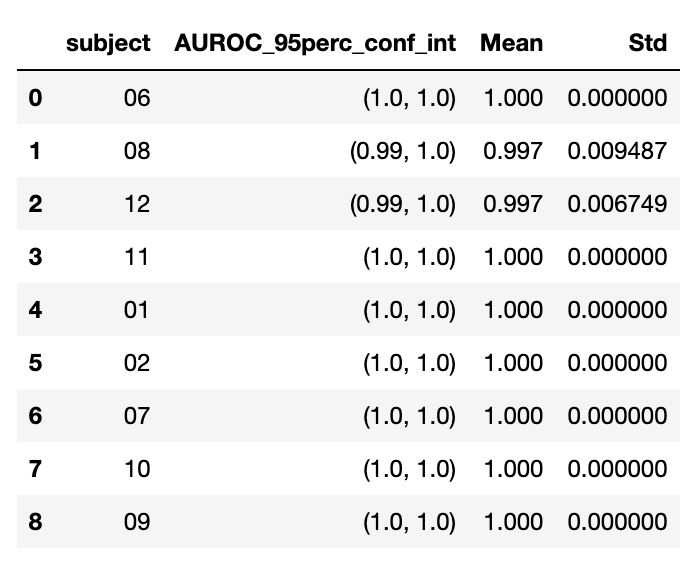
**

##### **SI Table 9.** Logistic regression one-versus-rest models built to classify one individual’s HB1 phageprints after ~600 dominant OTUs were removed and the resulting OTU table was subsampled to contain only 2% of the total OTUs (~200). For each model type, 10 independent models based on 10 different train/test splits are built, and the 95% confidence intervals, mean and standard deviation for the Area Under the Precision Recall curve (AUPR) is reported in this table.


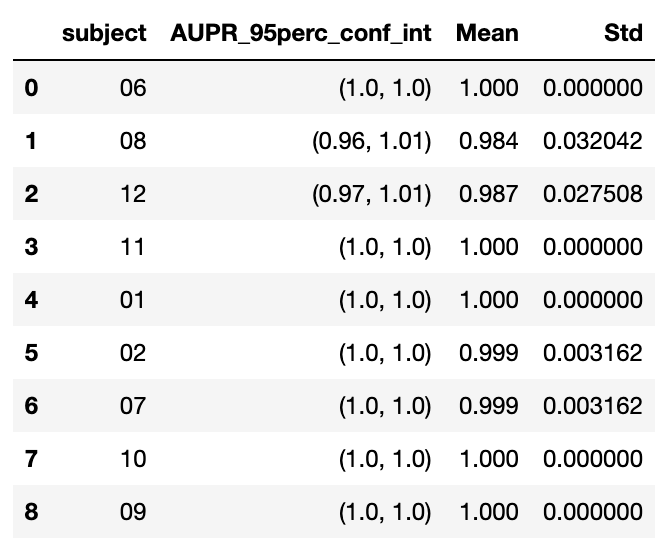


##### **SI Table 10.** Summary statistics for SI Figure 15. The absolute count and fraction of OTUs detected at least once in an individual are reported under the “count” and “frac_non_zero” columns, respectively. The mean, standard deviation, and median number of days OTUs are detected among other statistics are reported.


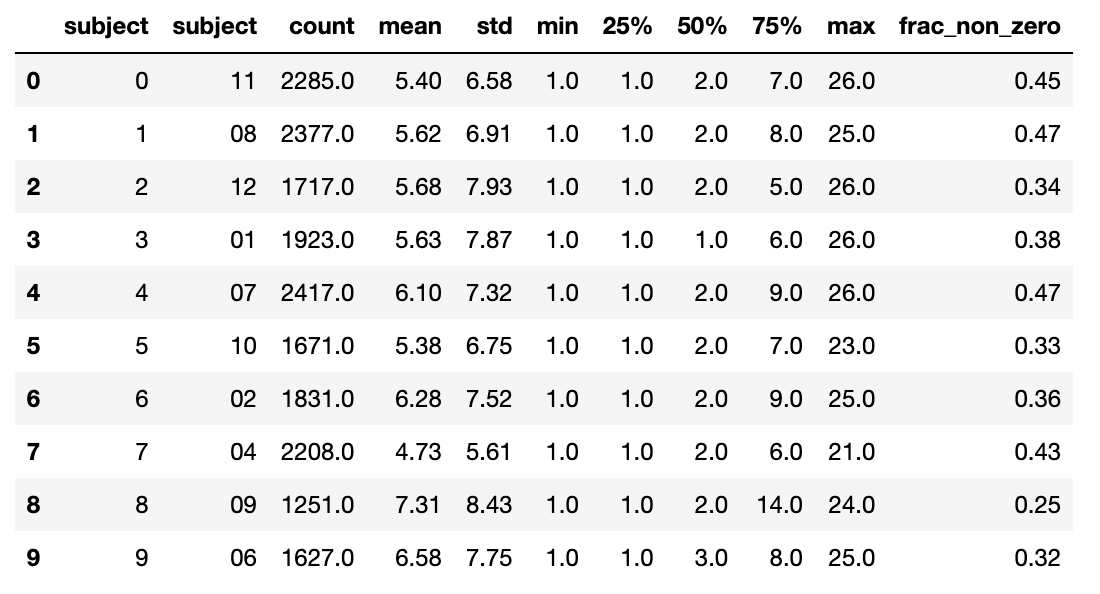

Supplement: Supplementary file 1 [file DataSheet1.docx]
